# Establishment and Validation of a Non-invasive Diagnostic Nomogram to Identify Heart Failure in Patients With Coronary Heart Disease

**DOI:** 10.3389/fcvm.2022.875702

**Published:** 2022-04-07

**Authors:** Juntao Tan, Yuxin He, Zhanbiao Li, Xiaomei Xu, Qinghua Zhang, Qian Xu, Lingqin Zhang, Shoushu Xiang, Xuewen Tang, Wenlong Zhao

**Affiliations:** ^1^Operation Management Office, Affiliated Banan Hospital of Chongqing Medical University, Chongqing, China; ^2^Department of Medical Administration, Affiliated Banan Hospital of Chongqing Medical University, Chongqing, China; ^3^Department of Gastroenterology, The Fifth People’s Hospital of Chengdu, Chengdu, China; ^4^Department of Infectious Diseases, The First Affiliated Hospital of Chongqing Medical University, Chongqing, China; ^5^Department of Science and Education, Affiliated Banan Hospital of Chongqing Medical University, Chongqing, China; ^6^College of Medical Informatics, Chongqing Medical University, Chongqing, China; ^7^Medical Data Science Academy, Chongqing Medical University, Chongqing, China; ^8^Library, Chongqing Medical University, Chongqing, China; ^9^Department of Biomedical Equipment, People’s Hospital of Chongqing Bishan District, Chongqing, China; ^10^Department of Cardiology, Affiliated Banan Hospital of Chongqing Medical University, Chongqing, China

**Keywords:** coronary heart disease, heart failure, prediction model, nomogram, calculator tool

## Abstract

**Background:**

Heart failure (HF) is an end-stage manifestation of and cause of death in coronary heart disease (CHD). The objective of this study was to establish and validate a non-invasive diagnostic nomogram to identify HF in patients with CHD.

**Methods:**

We retrospectively analyzed the clinical data of 44,772 CHD patients from five tertiary hospitals. Univariate logistic regression analyses and least absolute shrinkage and selection operator (LASSO) regression analyses were used to identify independent factors. A nomogram based on the multivariate logistic regression model was constructed using these independent factors. The concordance index (C-index), receiver operating characteristic (ROC) curves, calibration curves, decision curve analysis (DCA), and clinical impact curves (CIC) were used to evaluate the predictive accuracy and clinical value of this nomogram.

**Results:**

The predictive factors in the multivariate model included hypertension, age, and the total bilirubin, uric acid, urea nitrogen, triglyceride, and total cholesterol levels. The area under the curve (AUC) values of the nomogram in the training set, internal validation set, external validation set1, and external validation set2 were 0.720 (95% CI: 0.712–0.727), 0.723 (95% CI: 0.712–0.735), 0.692 (95% CI: 0.674–0.710), and 0.655 (95% CI: 0.634–0.677), respectively. The calibration curves indicated that the nomogram had strong calibration. DCA and CIC indicated that the nomogram can be used as an effective tool in clinical practice.

**Conclusion:**

The developed predictive model combines the clinical and laboratory factors of patients with CHD and is useful in individualized prediction of HF probability for clinical decision-making during treatment and management.

## Introduction

With the development of the social economy, changes in dietary structures, and the trend of aging populations, coronary heart disease (CHD) has become one of the main causes of harm to human health (1). It led to around 18 million (roughly one-third of) deaths worldwide in the year 2016 (2). The 2018 Cardiovascular Diseases Report in China estimated that 290 million Chinese people suffer from cardiovascular diseases, including 11 million suffering from CHD, accounting for the first cause of death (3). In addition to its effects on human health, CHD also imposes a heavy burden on society and families because of the reduction in the labor force and the high medical expenses. Due to the high morbidity, disability, and fatality rates of CHD, its prevention and treatment have been the focus of research both home and abroad (4, 5).

Heart failure (HF) is a clinical syndrome caused by defects in the structure and function of the myocardium, which result in impaired ventricular filling and/or ejection function (6). HF represents the ultimate pathway of numerous heart diseases (7). The incidence of HF in industrialized countries has been reported to be approximately 0.4–2.2%, with 500,000–600,000 events diagnosed each year (8, 9). The mortality rate in HF patients is high, as shown in the MAGGIC meta-analysis, which included individual data for 39,372 patients and reported that 40.2% of the patients died during a median follow-up period of 2.5 years (10). The incidence and prevalence of HF are projected to increase further in China, as in many other low- and middle-income countries (11). In China, the in-hospital mortality rates for chronic and acute HF patients have been reported to be 1.9 and 10.8%, respectively (12).

CHD has been proven to be the main etiological factor for HF (13). Uncontrolled CHD will lead to HF, thereby increasing the difficulty of clinical treatment and threatening the safety of patients (14). Thus, there is an urgent need to strengthen research on the clinical treatment of CHD and HF, identify the most optimal safe and effective treatment plans for patients through multi-exploration, maximize the clinical treatment effect, and reduce the threat of the disease to the health and safety of patients.

Although many studies have discussed the morbidity, diagnosis, and treatment of HF, its early diagnosis still presents some difficulties. This study aimed to establish a simple and effective method for diagnosing HF in patients with CHD on the basis of large samples and common clinical indicators of CHD. In addition, we developed an online application based on a nomogram to diagnose HF in patients with CHD. This study can serve as a reference for early clinical screening of CHD patients at a high risk of HF and facilitate early initiation of predictive interventions.

## Materials and Methods

### Data Source

The study data were obtained from the electronic medical records of five tertiary hospitals on the Big Data Platform of the Medical Data Research Institute of Chongqing Medical University. The platform includes more than 40 million electronic medical records from seven tertiary hospitals in Chongqing, with the data deidentified to ensure patient privacy. This study was approved by the Ethics Committee of the Affiliated Banan Hospital of Chongqing Medical University. Written informed consent for participation was not required for this study due to its retrospective design, and the study was conducted in accordance with national legislation and institutional requirements.

### Definition

The target population was defined as patients who were initially diagnosed with CHD on the basis of clinical, biochemical, and imaging findings or past medical records, and in accordance with the “Chinese guidelines for the management of coronary heart disease” (15). In other cases, the diagnosis of HF was based on criteria from the consensus recommendation of the Asia Pacific Heart Rhythm Society of HF (16).

### Inclusion and Exclusion Criteria

The inclusion criteria were as follows: (i) data obtained from 2015 to 2021, (ii) patients aged ≥ 18 years, and (iii) hospitalization(s) with CHD. The exclusion criteria were as follows: (i) patients with cancer, mental illness, or other serious complications and (ii) patients with > 30% missing data. The study selection process is depicted in Supplementary Figure 1.

### Data Collection

On the basis of previous studies, 26 possible risk factors for predicting HF were selected, namely, diabetes, hypertension, smoking status, drinking status, sex, age, diastolic blood pressure (DBP), white blood cell (WBC) count, red blood cell (RBC) count, neutrophil-to-lymphocyte ratio (NLR), lymphocyte-to-monocyte ratio (LMR), platelet-to-lymphocyte ratio (PLR), serum creatinine (SCr), total bilirubin (TB), uric acid (UA) level, urea nitrogen (UN), total protein (TP), alkaline phosphatase (ALP), low-density lipoprotein (LDL), hemoglobin (HGB), blood glucose (BG), aspartate aminotransferase (AST), triglyceride (TG), total cholesterol (TC), high-density lipoprotein (HDL), and γ-glutamyltransferase (GGT) levels.

### Statistical Analyses

The data included in this study were divided into three groups: The data from hospital A-D for 2015 to 2019 were divided into the training set and internal validation set in a ratio of 7:3; the data from hospital A-D for 2020 and 2021 were used as external validation set1; and the data from hospital E were used as external validation set2.

Statistical analyses were performed using SPSS 22.0 and R (version 4.0.2, Vienna, Austria). Univariate logistic regression analyses and least absolute shrinkage and selection operator (LASSO) regression analyses were performed to identify independent factors. A nomogram was constructed on the basis of these independent factors. The discriminatory values of the models were assessed on the basis of the concordance index (C-index). The area under the curve (AUC) of the receiver operating characteristic (ROC) curve was used to evaluate the accuracy of the nomogram. Calibration curves were established to evaluate the calibration of the nomogram. In addition, decision curve analysis (DCA) and clinical impact curve (CIC) were used to demonstrate the clinical benefit of the nomogram. The multiple imputation method was used to fill in the missing continuous variables. The enumerative data were expressed as rate and percentage, and the chi-square test was used for comparisons between groups. The quantitative variables did not show a normal distribution and were represented by median and interquartile range [M (Q25-Q75)], and comparisons between groups were performed with the Mann-Whitney *U* test. All statistical analyses were two-sided, and statistical significance was set at *P* < 0.05.

## Results

### Patient Characteristics

A total of 44,772 CHD patients were included in this study. They were divided into the training set (*n* = 26,269), internal validation set (*n* = 11,259), external validation set1 (*n* = 4,365), and external validation set2 (*n* = 2,879). The Mann-Whitney *U* test showed no significant differences in any of the missing variables before and after multiple imputation in the training and internal validation sets (Table 1). We also observed no significant differences in any of the missing variables before and after multiple imputation in external validation set1 and external validation set2 (Supplementary Table 1 and Table 2). The demographic and clinical characteristics of the training and validation sets are presented in Table 2. No significant differences were observed in any of the variables between the training and internal validation sets. The demographic and clinical characteristics of the training and external validation sets are listed in Supplementary Tables 3, 4.

**TABLE 1:** Comparison of continuous variables in the training and internal validation sets before and after multiple imputation.

Variables	Before interpolation	After interpolation	*P*-values
DBP (IQR, mmHg)	80.00 (71.00, 89.00)	80.00 (71.00, 89.00)	0.978
GGT (IQR, IU/L)	27.00 (18.00, 49.00)	27.00 (18.00, 49.00)	0.920
WBC (IQR, ×10^9^/L)	6.60 (5.30, 8.50)	6.60 (5.30, 8.50)	0.424
NLR (IQR)	3.36 (2.24,5.89)	3.37 (2.24, 5.94)	0.693
PLR (IQR)	137.61 (100.65, 197.57)	137.90 (100.64, 198.46)	0.572
LMR (IQR)	3.33 (2.11, 4.92)	3.31 (2.09, 4.91)	0.171
SCr (IQR, μmol/l)	70.00 (56.30, 88.83)	70.00 (56.40, 89.17)	0.173
TB (IQR, μ/l)	10.60 (7.60,14.60)	10.60 (7.60,14.70)	0.666
UA (IQR, μ/L)	334.40 (268.20, 412.20)	334.40 (268.00, 412.70)	0.919
UN (IQR, mmol/L)	6.08 (4.84, 7.89)	6.09 (4.85, 7.91)	0.285
TP (IQR, g/L)	67.60 (62.70, 72.50)	67.60 (62.70, 72.50)	0.865
ALP (IQR, IU/L)	75.90 (62.00, 93.00)	75.36 (62.00, 93.00)	0.758
LDL (IQR, mmol/L)	2.32 (1.78, 2.93)	2.32 (1.78, 2.92)	0.959
HGB (IQR, g/L)	130.00 (117.00, 142.00)	130.00 (117.00, 142.00)	0.813
RBC (IQR, ×10^12^/L)	4.30 (3.88, 4.69)	4.30 (3.88, 4.69)	0.686
BG (IQR, mmol/L)	5.91 (5.09, 7.84)	5.93 (5.10, 7.90)	0.138
AST (IQR, IU/L)	22.00 (17.12, 30.00)	22.00 (17.19, 30.00)	0.413
TGs (IQR, mmol/L)	1.28 (0.93, 1.84)	1.27 (0.92,1.84)	0.231
TC (IQR, mmol/L)	4.27 (3.55, 5.06)	4.28 (3.56, 5.06)	0.792
HDL (IQR, mmol/L)	1.19 (0.97, 1.44)	1.19 (0.97, 1.44)	0.753

*DBP, diastolic blood pressure; GGT,γ-glutamyltransferase; WBC, white blood cell; NLR, neutrophil-to-lymphocyte ratio; PLR, platelet-lymphocyte ratio; LMR, lymphocyte-to-monocyte ratio; SCr, serum creatinine; TB, total bilirubin; UA, uric acid; UN, urea nitrogen; TP, total protein; ALP, alkaline phosphatase; LDL, low-density lipoprotein; HGB, hemoglobin; RBC, red blood cell; BG, blood glucose; AST, aspartate aminotransferase; TGs, triglycerides; TC, total cholesterol; HDL, high-density lipoprotein; IQR, interquartile range.*

**TABLE 2:** Demographic and clinical characteristics of the training and internal validation sets.

Variables	Training set (*N* = 26,269)	Internal validation set (*N* = 11,259)	*P-*values
Diabetes (*n*, %)	6,194 (23.58%)	2,619 (23.26%)	0.514
Hypertension (*n*, %)	10,161 (38.68%)	4,330 (38.46%)	0.694
Smoking status (*n*, %)	9,044 (34.43%)	3,909 (34.72%)	0.596
Drinking status (*n*, %)	7,103 (27.04%)	3,083 (27.38%)	0.502
Sex (male, *n*, %)	13,031 (49.61%)	5,602 (49.76%)	0.799
Age (IQR, years)	70.00 (61.00, 78.00)	70.00 (61.00, 78.00)	0.574
DBP (IQR, mmHg)	80.00 (71.00, 89.00)	80.00 (71.00, 89.00)	0.720
GGT (IQR, IU/L)	27.00 (18.00, 49.00)	27.20 (18.00, 48.00)	0.687
WBC (IQR, ×10^9^/L)	6.60 (5.30, 8.50)	6.60 (5.30, 8.53)	0.721
NLR (IQR)	3.37 (2.24, 5.93)	3.36 (2.22, 5.98)	0.786
PLR (IQR)	138.13 (100.91, 200.00)	137.39 (100.00, 196.25)	0.272
LMR (IQR)	3.30 (2.08, 4.88)	3.34 (2.11, 5.00)	0.058
SCr (IQR, μmol/l)	70.00 (56.30, 89.60)	70.00 (56.70, 88.79)	0.561
TB (IQR, μmol/l)	10.60 (7.60, 14.70)	10.60 (7.60, 14.70)	0.541
UA (IQR, μmol/L)	334.80 (268.30, 414.00)	333.90 (267.60, 409.80)	0.125
UN (IQR, mmol/L)	6.10 (4.85, 7.92)	6.05 (4.83, 7.91)	0.351
TP (IQR, g/L)	67.60 (62.68, 72.50)	67.50 (62.70, 72.50)	0.612
ALP (IQR, IU/L)	75.27 (62.00, 93.00)	75.84 (62.00, 93.00)	0.788
LDL (IQR, mmol/L)	2.32 (1.78, 2.93)	2.32 (1.77, 2.92)	0.567
HGB (IQR, g/L)	130.00 (117.00, 142.00)	130.00 (117.00, 142.00)	0.903
RBC (IQR, ×10^12^/L)	4.30 (3.88, 4.70)	4.30 (3.88, 4.69)	0.837
BG (IQR, mmol/L)	5.95 (5.10, 7.91)	5.90 (5.10, 7.87)	0.162
AST (IQR, IU/L)	22.00 (17.20, 30.00)	22.00 (17.17, 30.00)	0.954
TGs (IQR, mmol/L)	1.27 (0.92, 1.84)	1.28 (0.92, 1.84)	0.996
TC (IQR, mmol/L)	4.27 (3.56, 5.06)	4.28 (3.55, 5.06)	0.887
HDL (IQR, mmol/L)	1.19 (0.97, 1.44)	1.19 (0.97, 1.44)	0.847

*DBP, diastolic blood pressure; GGT, γ-glutamyltransferase; WBC, white blood cell; NLR, neutrophil-to-lymphocyte ratio; PLR, platelet-lymphocyte ratio; LMR, lymphocyte-to-monocyte ratio; SCr, serum creatinine; TB, total bilirubin; UA, uric acid; UN, urea nitrogen; TP, total protein; ALP, alkaline phosphatase; LDL, low-density lipoprotein; HGB, hemoglobin; RBC, red blood cell; BG, blood glucose; AST, aspartate aminotransferase; TGs, triglycerides; TC, total cholesterol; HDL, high-density lipoprotein; IQR, interquartile range.*

### Selection of Predictors for Heart Failure in Coronary Heart Disease Patients

**FIGURE 1:**
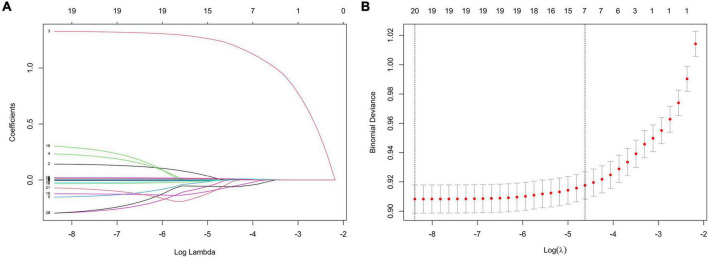
Features selection by LASSO. **(A)** LASSO coefficients profiles (y-axis) of the 20 features. The upper x-axis shows the average numbers of predictors and the lower x-axis shows the log(λ). **(B)** Fivefold cross-validation for tuning parameter selection in the LASSO model.

In the training set, statistically significant differences were observed in diabetes, hypertension, smoking status, drinking status, sex, age, and most laboratory variables between the HF and non-HF groups (Table 3); LMR, TP, ALP, BG, and AST were within the normal reference value range in the vast majority of patients included in this study, with no significant difference between groups. The LASSO coefficient profiles with non-zero coefficients determined by the optimal lambda (λ) are shown in Figures 1A,B. λ is the regularization parameter in LASSO, and the optimal value could be obtained from the 10-fold cross-validation. When log (λ) = –4.7 (minMSE + 1SE), seven predictive factors were selected: hypertension (OR = 3.912, 95% CI = 3.667–4.175), age (OR = 1.015, 95% CI = 1.013–1.018), TB (OR = 1.018, 95% CI = 1.014–1.023), UA (OR = 1.002, 95% CI = 1.001–1.003), UN (OR = 1.016, 95% CI = 1.007–1.025), TGs (OR = 0.878, 95% CI = 0.843–0.914), and TC (OR = 0.902, 95% CI = 0.873–0.931) (Table 4). Additionally, the regression coefficients of the other 19 indicators were compressed to zero and excluded.

**TABLE 3:** Univariate analyses of variables associated with HF.

Variables	HF (*N* = 5,384)	Non-HF (*N* = 20,885)	*P*-values
Diabetes (*n*, %)	1,541 (28.62%)	4,653 (22.28%)	<0.001
Hypertension (*n*, %)	3,528 (65.53%)	6,633 (31.76%)	<0.001
Smoking status (*n*, %)	1,786 (33.17%)	7,258 (34.75%)	0.031
Drinking status (*n*, %)	1,285 (23.87%)	5,818 (27.86%)	<0.001
Sex (male, *n*, %)	2,559 (47.53%)	10,472 (50.14%)	<0.001
Age (IQR, years)	73.00 (64.00, 81.00)	69.00 (60.00, 77.00)	<0.001
DBP (IQR, mmHg)	78.00 (70.00, 88.00)	80.00 (71.00, 89.00)	<0.001
GGT (IQR, IU/L)	28.00 (18.00, 52.00)	27.00 (18.00, 48.00)	<0.001
WBC (IQR, ×10^9^/L)	6.52 (5.24, 8.29)	6.60 (5.30, 8.60)	<0.001
NLR (IQR)	3.44 (2.33, 5.64)	3.35 (2.22, 6.01)	0.138
PLR (IQR)	137.01 (100.10, 197.39)	138.42 (101.08, 200.79)	0.041
LMR (IQR)	3.29 (2.12, 4.83)	3.31 (2.07, 4.89)	0.704
SCr (IQR, μmol/l)	74.90 (59.40, 97.73)	69.00 (56.00, 87.50)	<0.001
TB (IQR, μmol/l)	11.10 (8.10, 15.40)	10.40 (7.50, 14.40)	<0.001
UA (IQR, μmol/L)	359.80 (288.38, 443.70)	329.00 (264.00, 406.20)	<0.001
UN (IQR, mmol/L)	6.48 (5.11, 8.66)	6.00 (4.80, 7.74)	<0.001
TP (IQR, g/L)	67.50 (62.70, 72.50)	67.65 (62.60, 72.50)	0.886
ALP (IQR, IU/L)	75.89 (62.00, 93.00)	75.00 (62.00, 93.00)	0.974
LDL (IQR, mmol/L)	2.26 (1.74,2.86)	2.35 (1.80, 2.94)	<0.001
HGB (IQR, g/L)	128.00 (115.00, 140.00)	130.00 (117.00, 142.00)	<0.001
RBC (IQR, ×10^12^/L)	4.23 (3.81, 4.64)	4.31 (3.89, 4.71)	<0.001
BG (IQR, mmol/L)	5.99 (5.13, 7.76)	5.93 (5.10, 7.95)	0.634
AST (IQR, IU/L)	22.00 (17.70, 30.00)	22.00 (17.00, 30.00)	0.294
TGs (IQR, mmol/L)	1.21 (0.89, 1.71)	1.29 (0.93, 1.88)	<0.001
TC (IQR, mmol/L)	4.09 (3.41,4.87)	4.32 (3.60,5.10)	<0.001
HDL (IQR, mmol/L)	1.14 (0.93, 1.38)	1.20 (0.98, 1.46)	<0.001

*HF, Heart failure; DBP, diastolic blood pressure; GGT, γ-glutamyltransferase; WBC, white blood cell; NLR, neutrophil-to-lymphocyte ratio; PLR, platelet-lymphocyte ratio; LMR, lymphocyte-to-monocyte ratio; SCr, serum creatinine; TB, total bilirubin; UA, uric acid; UN, urea nitrogen; TP, total protein; ALP, alkaline phosphatase; LDL, low-density lipoprotein; HGB, hemoglobin; RBC, red blood cell; BG, blood glucose; AST, aspartate aminotransferase; TGs, triglycerides; TC, total cholesterol; HDL, high-density lipoprotein; IQR, interquartile range.*

**TABLE 4:** Results of multivariate logistic regression model.

Variables	β	SE	OR (95%CI)	*P*-values
Hypertension	1.364	0.033	3.912 (3.667, 4.175)	<0.001
Age	0.015	0.001	1.015 (1.013, 1.018)	<0.001
TB	0.018	0.002	1.018 (1.014, 1.023)	<0.001
UA	0.002	0.001	1.002 (1.001, 1.003)	<0.001
UN	0.016	0.004	1.016 (1.007, 1.025)	<0.001
TGs	–0.130	0.020	0.878 (0.843, 0.914)	<0.001
TC	–0.103	0.016	0.902 (0.873, 0.931)	<0.001

*TB, total bilirubin; UA, uric acid; UN, urea nitrogen; TGs, triglycerides; TC, total cholesterol; SE, Standard Error; OR, odds ratio; CI, Confidence Interval.*

### Nomogram Construction and Performance

**FIGURE 2:**
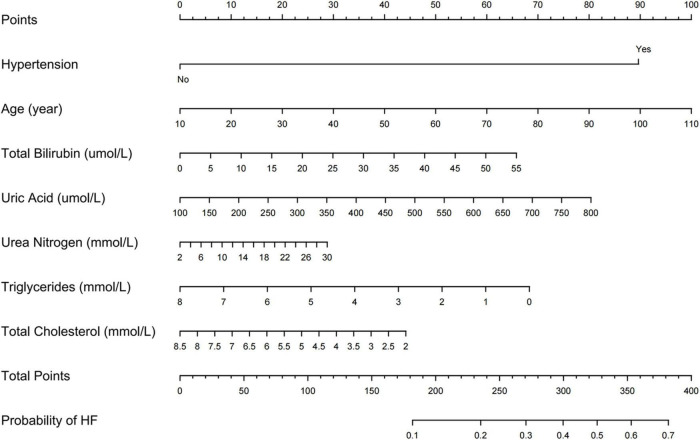
Nomogram predicting HF in patients with CHD.

**FIGURE 3:**
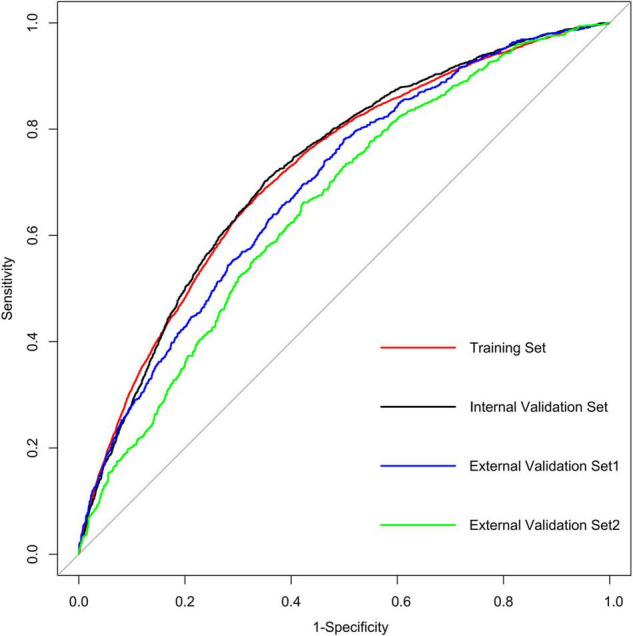
AUC of the ROC curve in training set, internal validation set, external validation set1 and external validation set2.

**FIGURE 4:**
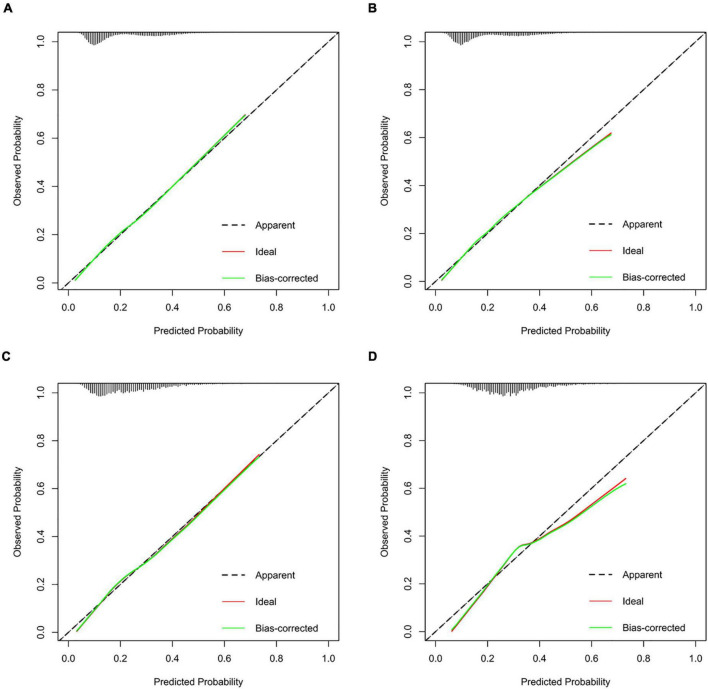
Calibration curves of the HF incidence risk nomogram prediction in training set **(A)**, internal validation set **(B)**, external validation set1 **(C)**, and external validation set2 **(D)**.

We further incorporated the independent predictors mentioned above into a nomogram to predict the probability of HF (Figure 2). Each parameter was assigned an exact point. A higher total sum of the designated points in the nomogram indicated a higher risk of HF. A C-index of 0.720 (95% CI: 0.712–0.727) was calculated as the discriminative value for this nomogram, indicating good predictive power (Table 5 and Figure 3). Moreover, the calibration curves (bootstraps = 1,000) of the training set indicated that the nomogram had strong calibration (Figure 4A). The details of internal validation set, external validation set1 and external validation set2 were shown in Figures 4B–D, respectively.

**TABLE 5:** Detailed performance metrics for the four models.

Models	Sensitivity	Specificity	PPV	NPV	AUC (95%CI)
Training set	0.341	0.888	0.675	0.664	0.720 (0.712–0.727)
Internal validation set	0.333	0.897	0.702	0.649	0.723 (0.712–0.735)
External validation set1	0.315	0.885	0.779	0.501	0.692 (0.674–0.710)
External validation set2	0.391	0.806	0.661	0.577	0.655 (0.634–0.677)

*PPV, positive predictive value; NPV, negative predictive value; AUC, area under the curve; CI, Confidence Interval.*

### Clinical Utility of the Nomogram

**FIGURE 5:**
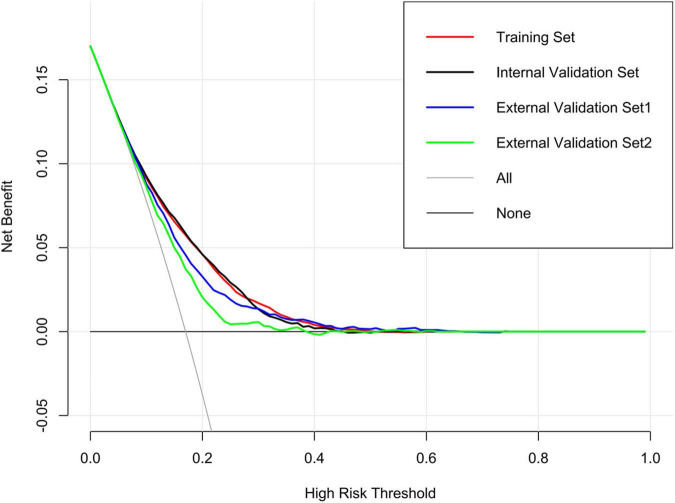
Decision curve analysis (DCA) of the nomogram in training set, internal validation set, external validation set1 and external validation set2.

**FIGURE 6:**
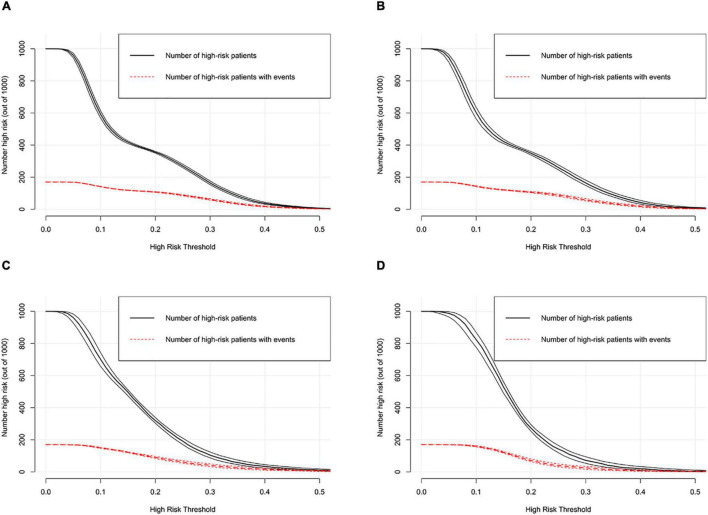
Clinical impact curve of the nomogram in training set **(A)**, internal validation set **(B)**, external validation set1 **(C)** and external validation set2 **(D)**.

DCA was performed to investigate the net benefits of this predictive model (Figure 5). The results suggested that when the nomogram-predicted probability of HF was < 49%, the nomogram provided additional value relative to the treat-all-patients scheme or the treat-none scheme, suggesting that the nomogram was clinically useful. In addition, the CIC was further drawn according to DCA to evaluate the clinical impact of the nomogram and thereby intuitively understand its substantive value. In the training set (Figure 6A), the CIC showed that the nomogram had significant predictive power. Figure 6A shows the estimated number of patients expected to reach HF at each risk threshold and the number of patients experiencing HF. When the risk threshold exceeded 20%, the estimated number of patients was closer to the actual number of patients. The details of internal validation set, external validation set1 and external validation set2 were shown in Figures 6B–D, respectively.

### Construction of Web App to Easily Access the Nomogram

**FIGURE 7:**
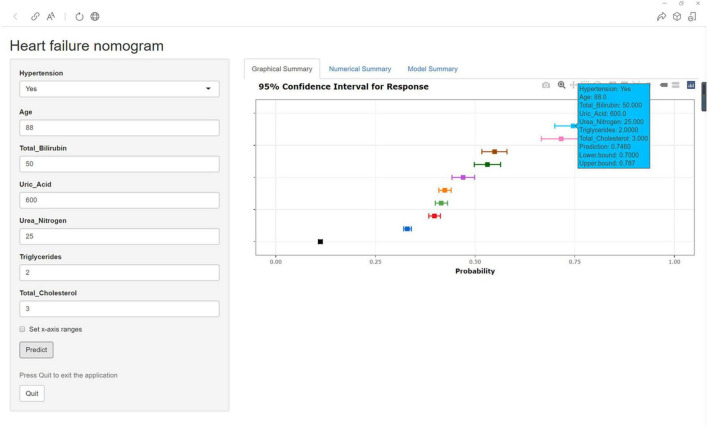
An example of HF prediction using the nomogram in patients with CHD via a link.

Finally, the nomogram was made accessible to medical staff via a link. The algorithm automatically calculates a patient’s probability of HF^1^. The scoring system enables early identification of high-risk patients, facilitating adoption of appropriate treatment measures. For example, when a patient has hypertension, an age of 88 years, TB of 50.00 μmol/l, UA of 600.00 μmol/l, UN of 25.00 mmol/l, TGs of 2.00 mmol/l, and TC of 3.00 mmol/l, the probability of HF would be 0.746 (95% CI: 0.700–0.787) (Figure 7).

## Discussion

HF poses a serious threat to the quality of life and survival of patients with CHD. Thus, early detection of the risk of HF in patients with CHD is helpful in improving their quality of life. In this study, a multivariate predictive model to detect HF in patients with CHD was developed and validated. The LASSO logistic regression model selected the following seven predictors: hypertension, age, and TB, UA, UN, TG, and TC levels. The nomogram application revealed good discrimination in distinguishing CHD patients with HF. The calibration curves indicated that the nomogram had strong calibration, and DCA and CIC indicated that the nomogram can be used as an effective tool in clinical practice.

Several studies have shown that longstanding hypertension ultimately leads to HF; conversely, the absence of hypertension in middle age is associated with lower risks of incident HF across the remaining life course (17–19). In the Framingham Heart Study cohort consisting of a total population of 5,143 participants, hypertension antedated the development of HF in 91% of all newly diagnosed HF patients over 20 years of follow-up (mean, 14.1 years) (20). In our study, in comparison to patients without hypertension, those with hypertension showed a 3.912–fold higher risk of HF. Long-term persistent hypertension can cause pathological cardiomyocyte hypertrophy and myocardial injury, resulting in myocardial hypertrophy, eccentric myocardial hypertrophy, cardiac chamber enlargement, and a series of myocardial remodeling processes, ultimately leading to HF (21). Recent studies have indicated that initial therapy with most antihypertensive drugs can decelerate the transition from hypertension to HF, although the effects of these drugs are not equal in this regard (22–24). Therefore, clinicians can perform personalized antihypertensive treatment planning based on the patient’s actual situation.

Our study showed that an elevated TB level was a risk factor for HF in patients with CHD, which is consistent with the findings of previous studies (25, 26). An increase in TB levels reflects liver damage and a decline in liver synthetic function (27). Patients with HF often experience systemic congestion at the end stage, which prevents the hepatic venous blood from returning to the patient’s body, resulting in liver congestion. Congestion in the liver reduces the effect of bilirubin, and simultaneously, the TB produced by human metabolism cannot be converted into bilirubin, resulting in an increase in the TB level in the human body. Our study also showed that an elevated UA level was a risk factor for HF in patients with CHD. UA is the end-product of purine catabolism *in vivo*. Hyperuricemia has been shown to be closely related to the occurrence and development of chronic HF (28). The higher the UA value, the more obvious is the mortality of patients with HF. The serum UA level can be used as a predictor of the prognosis of patients with HF (29, 30). Peter et al. found that an increase in UA level is related to the severity of HF and positively correlated with an increase in the NYHA level (31). In addition, TB and UA can be used as biochemical indicators reflecting the oxidation and antioxidant systems in patients with CHD, and they may play important roles in the diagnosis and treatment of CHD. Therefore, these indicators should be regularly monitored in patients with CHD in clinical practice, which will help clinicians obtain a correct understanding of the risk of HF and disease progression.

UN is a protein metabolite of the human body that is synthesized in the liver and excreted by the kidneys. Therefore, UN level represents a balance between urea production and renal excretion and is an important marker of renal damage. In the past, the UN level was primarily used to reflect renal function. However, Aronson et al. first studied the value of the UN level for prognostication of patients admitted for acute decompensated HF (32). This observation was validated and generalized by other investigators who found that an elevated UN level was an independent predictor of adverse outcomes in patients with acute and chronic HF (33–35). In addition, other studies have shown that baseline UN levels represent the severity of HF and are a better prognostic marker of adverse clinical events than is creatinine or estimated glomerular filtration rate (36, 37). We also found that age and TG and TC levels were discussed in a previous study of influencing factors (38–40).

Taken together, the findings suggest that hypertension, age, and TB, UA, UN, TG, and TC levels are useful clinical parameters for predicting HF in patients with CHD, and can facilitate early identification of patients with a high risk of HF. However, this study also had several limitations. First, the retrospective nature of the investigation may have introduced selection bias. However, a multicenter and relatively large training set was used to build the models, which were further subjected to temporal and spatial validation. Second, data for social support, socioeconomic status, and other important factors were not available. Further research is warranted to explore the impact of these important indicators.

## Conclusion

This study developed and validated a diagnostic model based on the common clinical features of patients with CHD. Hypertension, age, and TB, UA, UN, TG, and TC levels were independent predictors of HF in patients with CHD. The nomogram constructed on the basis of these factors provided good discrimination to distinguish individuals with HF among patients with CHD. The developed web calculator tool makes the application of the nomogram more convenient for clinicians.

## Data Availability Statement

The raw data supporting the conclusions of this article will be made available by the authors, without undue reservation.

## Ethics Statement

This study was approved by the Ethics Committee of the Affiliated Banan Hospital of Chongqing Medical University. Written informed consent for participation was not required for this study due to its retrospective design, and the study was undertaken in accordance with national legislation and institutional requirements.

## Author Contributions

JT and WZ designed the research. JT, ZL, QZ, LZ, and XX collected and organized data. JT, QX, SX, and YH analyzed the data. JT drafted the manuscript. XT and WZ contributed to the critical revision of the manuscript. All authors contributed to the manuscript and approved the submitted version.

## Conflict of Interest

The authors declare that the research was conducted in the absence of any commercial or financial relationships that could be construed as a potential conflict of interest.

## Publisher’s Note

All claims expressed in this article are solely those of the authors and do not necessarily represent those of their affiliated organizations, or those of the publisher, the editors and the reviewers. Any product that may be evaluated in this article, or claim that may be made by its manufacturer, is not guaranteed or endorsed by the publisher.

## Footnotes

1
https://cytjt007.shinyapps.io/dynnomapp_heart_failure/

## References

[1] Shaya GE Leucker TM Jones SR Martin SS Toth PP. Coronary heart disease risk: low-density lipoprotein and beyond. *Trends Cardiovasc Med.* (2021) S1050-1738(21)00046-3. 10.1016/j.tcm.2021.04.002 33872757

[2] Nick T Lauren W Prachi B Kremlin W Mike R Melanie N. Cardiovascular disease in Europe: epidemiological update 2016. *Eur Heart J.* (2016) 37:3232–45. 10.1093/eurheartj/ehw334 27523477 PMC13480964

[3] Li-Yuan M Wei-Wei C Run-Lin G Li-Sheng L Man-Lu Z Yong-Jun W China cardiovascular diseases report 2018: an updated summary. *J Geriatr Cardiol.* (2020) 17:1–8. 10.11909/j.issn.1671-5411.2020.01.001 32133031 PMC7008101

[4] Jinhui S Shi Q. Expression of lncRNA-ANRIL before and after treatment and its predictive value for short-term survival in patients with coronary heart disease. *Biomed Res Int.* (2021) 2021:5431985. 10.1155/2021/5431985 34901274 PMC8664524

[5] Dan W Chang S Hua GZ Xi WY Tian LT Yue W Study of the mechanism of action of Guanxin Shutong capsules in the treatment of coronary heart disease based on metabolomics. *Front Pharmacol.* (2021) 12:650438. 10.3389/fphar.2021.650438 33867992 PMC8048374

[6] Butler J Skopicki H. Quest for pathophysiological understanding of heart failure with preserved ejection fraction: stiffened resolve, compliant approach. *J Am Coll Cardiol.* (2017) 70:149–51. 10.1016/j.jacc.2017.05.050 28683961

[7] Pagliaro BR Cannata F Stefanini GG Bolognese L. Myocardial ischemia and coronary disease in heart failure. *Heart Fail Rev.* (2020) 25:53–65. 10.1007/s10741-019-09831-z 31332663

[8] Go AS Mozaffarian D Roger VL Benjamin EJ Berry JD Blaha MJ Heart disease and stroke statistics–2014 update: a report from the American heart association. *Circulation.* (2014) 129:e28–292. 24352519 10.1161/01.cir.0000441139.02102.80 PMC5408159

[9] Biermann J Neumann T Angermann CE Düngen H-D Erbel R Herzog W Resource use and costs in systolic heart failure according to disease severity: a pooled analysis from the German competence network heart failure. *J Public Health.* (2012) 20:23–30. 10.1007/s10389-011-0452-0

[10] Pocock SJ Ariti CA McMurray JJ Maggioni A Køber L Squire IB Predicting survival in heart failure: a risk score based on 39 372 patients from 30 studies. *Eur Heart J.* (2013) 34:1404–13. 10.1093/eurheartj/ehs337 23095984

[11] Yang G Wang Y Zeng Y Gao GF Liang X Zhou M Rapid health transition in China, 1990-2010: findings from the global burden of disease study 2010. *Lancet.* (2013) 381:1987–2015. 10.1016/S0140-6736(13)61097-1 23746901 PMC7159289

[12] Wang N Zhao D Liu J Liu J Yu CM Wang W Impact of heart failure on in-hospital outcomes of acute coronary syndrome patients in China — results from the bridging the gap on CHD secondary prevention in China (BRIG) project. *Int J Cardiol.* (2012) 160:15–9. 10.1016/j.ijcard.2011.03.010 21453979

[13] Khatibzadeh S Farzadfar F Oliver J Ezzati M Moran A. Worldwide risk factors for heart failure: a systematic review and pooled analysis. *Int J Cardiol.* (2013) 168:1186–94. 10.1016/j.ijcard.2012.11.065 23201083 PMC3594565

[14] Hao Y Xie Y Li H Bao C. A3794 the safety and effect of small doses of perindopril on the patients of CHD with heart failure and low-normal blood pressure. *J Hypertens.* (2018) 36 (Suppl.):3.

[15] Jiang J Hong T Yu R Zhang Y Liu Z Huo Y. Knowledge of secondary prevention guidelines for coronary heart disease: results from a physicians’ survey in China. *Eur J Prev Cardiol.* (2012) 19:991–8. 10.1177/1741826711421299 21859776

[16] Lip GY Heinzel FR Gaita F Juanatey JR Le Heuzey JY Potpara T European heart rhythm association/heart failure association joint consensus document on arrhythmias in heart failure, endorsed by the heart rhythm society and the Asia Pacific heart rhythm society. *Europace.* (2016) 18:12–36. 10.1093/europace/euv191 26297713

[17] Messerli FH Rimoldi SF Bangalore S. The transition from hypertension to heart failure: contemporary update. *JACC Heart Fail.* (2017) 5:543–51. 10.1016/j.jchf.2017.04.012 28711447

[18] Mattias E Anna H Jan H Camilla H Thomas K Martin U The transition from hypertension to hypertensive heart disease and heart failure: the PREFERS hypertension study. *ESC Heart Fail.* (2020) 7:737–46. 10.1002/ehf2.12612 32073753 PMC7160482

[19] Kasiakogias A Rosei EA Camafort M Ehret G Kreutz R. Hypertension and heart failure with preserved ejection fraction: position paper by the European society of hypertension. *J Hypertens.* (2021) 39:1522–45. 10.1097/HJH.0000000000002910 34102660

[20] Levy D Larson MG Vasan RS Kannel WB Ho K. THe progression from hypertension to congestive heart failure. *JAMA.* (1996) 275:1557–62. 10.1001/jama.1996.03530440037034 8622246

[21] Michaels AT Peterson E Luzum J Gui H Pinto Y Sabbah HN Biomarker guided therapy for heart failure with mid-range EF. *J Cardiac Fail.* (2020) 26:S37. 10.1002/clc.23140 30578576 PMC6712323

[22] Costas T Gianfranco P Alberto Z. Effects of blood pressure-lowering treatment. 6. Prevention of heart failure and new-onset heart failure–meta-analyses of randomized trials. *J Hypertens.* (2016) 34:373–84. 10.1097/HJH.0000000000000848 26780184

[23] Chirag B Messerli FH Bernard K Ruilope LM Kazuomi K. Role of neprilysin inhibitor combinations in hypertension: insights from hypertension and heart failure trials. *Eur Heart J.* (2015) 36:1967–73. 10.1093/eurheartj/ehv142 25898846

[24] Bangalore S Fakheri R Toklu B Ogedegbe G Weintraub H Messerli FH Angiotensin-converting enzyme inhibitors or angiotensin receptor blockers in patients without heart failure? Insights from 254,301 Patients from randomized trials. *Mayo Clin Proc.* (2016) 91:51–60. 10.1016/j.mayocp.2015.10.019 26763511

[25] Okada A Sugano Y Nagai T Honda Y Iwakami N Nakano H Usefulness of the direct and/or total bilirubin to predict adverse outcomes in patients with acute decompensated heart failure. *Am J Cardiol.* (2017) 119:2035–41. 10.1016/j.amjcard.2017.03.033 28456315

[26] Zhang Y Zhang J Butler J Yang X Xie P Guo D Contemporary epidemiology, management, and outcomes of patients hospitalized for heart failure in china: results from the China heart failure (China-HF) registry. *J Cardiac Fail.* (2017) 23:868–75. 10.1016/j.cardfail.2017.09.014 29029965

[27] Shuichiro U Ryota H Takehisa Y Wataru I Takehito O Masakazu Y. Level of total bilirubin in the bile of the future remnant liver of patients with obstructive jaundice undergoing hepatectomy predicts postoperative liver failure. *J Hepato Biliary Pancreat Sci.* (2020) 27:614–21. 10.1002/jhbp.784 32506707

[28] Tamariz L Harzand A Palacio A Verma S Jones J Hare J Uric acid as a predictor of all-cause mortality in heart failure: a meta-analysis. *Congest Heart Fail.* (2011) 17:25–30. 10.1111/j.1751-7133.2011.00200.x 21272224

[29] Hamaguchi S Furumoto T Tsuchihashi-Makaya M Goto K Goto D Yokota T Hyperuricemia predicts adverse outcomes in patients with heart failure. *Int J Cardiol.* (2011) 151:143–7. 10.1016/j.ijcard.2010.05.002 20542341

[30] Wu AH Ghali JK Neuberg GW O’Connor CM Carson PE Levy WC. Uric acid level and allopurinol use as risk markers of mortality and morbidity in systolic heart failure. *Am Heart J.* (2010) 160:928–33. 10.1016/j.ahj.2010.08.006 21095282

[31] Peter O Martina O Jozef G Ivan T Jana Ko Mária O. Uric acid–a marker for systemic inflammatory response in patients with congestive heart failure? *Wien Klin Wochenschr.* (2002) 114:211–5. 12238311

[32] Aronson D Mittleman MA Burger AJ. Elevated blood urea nitrogen level as a predictor of mortality in patients admitted for decompensated heart failure. *Am J Med.* (2004) 116:466–73. 10.1016/j.amjmed.2003.11.014 15047036

[33] Chen C-Y Yoshida A Asakura M Hasegawa T Takahama H Amaki M Serum blood urea nitrogen and plasma brain natriuretic peptide and low diastolic blood pressure predict cardiovascular morbidity and mortality following discharge in acute decompensated heart failure patients. *Circ J.* (2012) 76:2372–9. 10.1253/circj.cj-12-0040 22785557

[34] Kajimoto K Minami Y Sato N Takano T. Serum sodium concentration, blood urea nitrogen, and outcomes in patients hospitalized for acute decompensated heart failure. *Int J Cardiol.* (2016) 222:195–201. 10.1016/j.ijcard.2016.07.255 27497094

[35] Khoury J Bahouth F Stabholz Y Elias A Mashiach T Aronson D Blood urea nitrogen variation upon admission and at discharge in patients with heart failure. *ESC Heart Fail.* (2019) 6:809–16. 10.1002/ehf2.12471 31199082 PMC6676277

[36] Klein L Massie BM Leimberger JD O’Connor CM Piña IL Adams KF Jr. Admission or changes in renal function during hospitalization for worsening heart failure predict postdischarge survival: results from the outcomes of a prospective trial of intravenous milrinone for exacerbations of chronic heart failure (OPTIME-CHF). *Circ Heart Fail.* (2008) 1:25–33. 10.1161/CIRCHEARTFAILURE.107.746933 19808267

[37] Nguyen T Soni A Phan R Bui TM Mai T Pinto D. GW27-e1214 new test to predict which heart failure patients will have BUN and creatinine increased by diuretics. *J Am Coll Cardiol.* (2016) 68:C151.

[38] Bosch L Assmann P Grauw W Schalk B Biermans M. Heart failure in primary care: prevalence related to age and comorbidity. *Prim Health Care Res Dev.* (2019) 20:e79. 10.1017/S1463423618000889 31868152 PMC6683237

[39] Varbo A Nordestgaard BG. Nonfasting triglycerides, low-density lipoprotein cholesterol, and heart failure risk: two cohort studies of 113 554 individuals. *Arterioscler Thromb Vasc Biol.* (2018) 38:464–72. 10.1161/ATVBAHA.117.310269 29097364

[40] Greene SJ Vaduganathan M Lupi L Ambrosy AP Mentz RJ Konstam MA Prognostic significance of serum total cholesterol and triglyceride levels in patients hospitalized for heart failure with reduced ejection fraction (from the EVEREST trial). *Am J Cardiol.* (2013) 111:574–81. 10.1016/j.amjcard.2012.10.042 23206923

