# Supplementary material for: Establishment and Validation of a Non-invasive Diagnostic Nomogram to Identify Heart Failure in Patients With Coronary Heart Disease
Source: Front Cardiovasc Med. 2022 Apr 7;9:875702. doi: 10.3389/fcvm.2022.875702 (PMC9021443; doi:10.3389/fcvm.2022.875702)
Supplement: Supplementary file 1 [file Data_Sheet_1.pdf]

Supplementary information

Figure S1 Flow of inclusions and exclusions

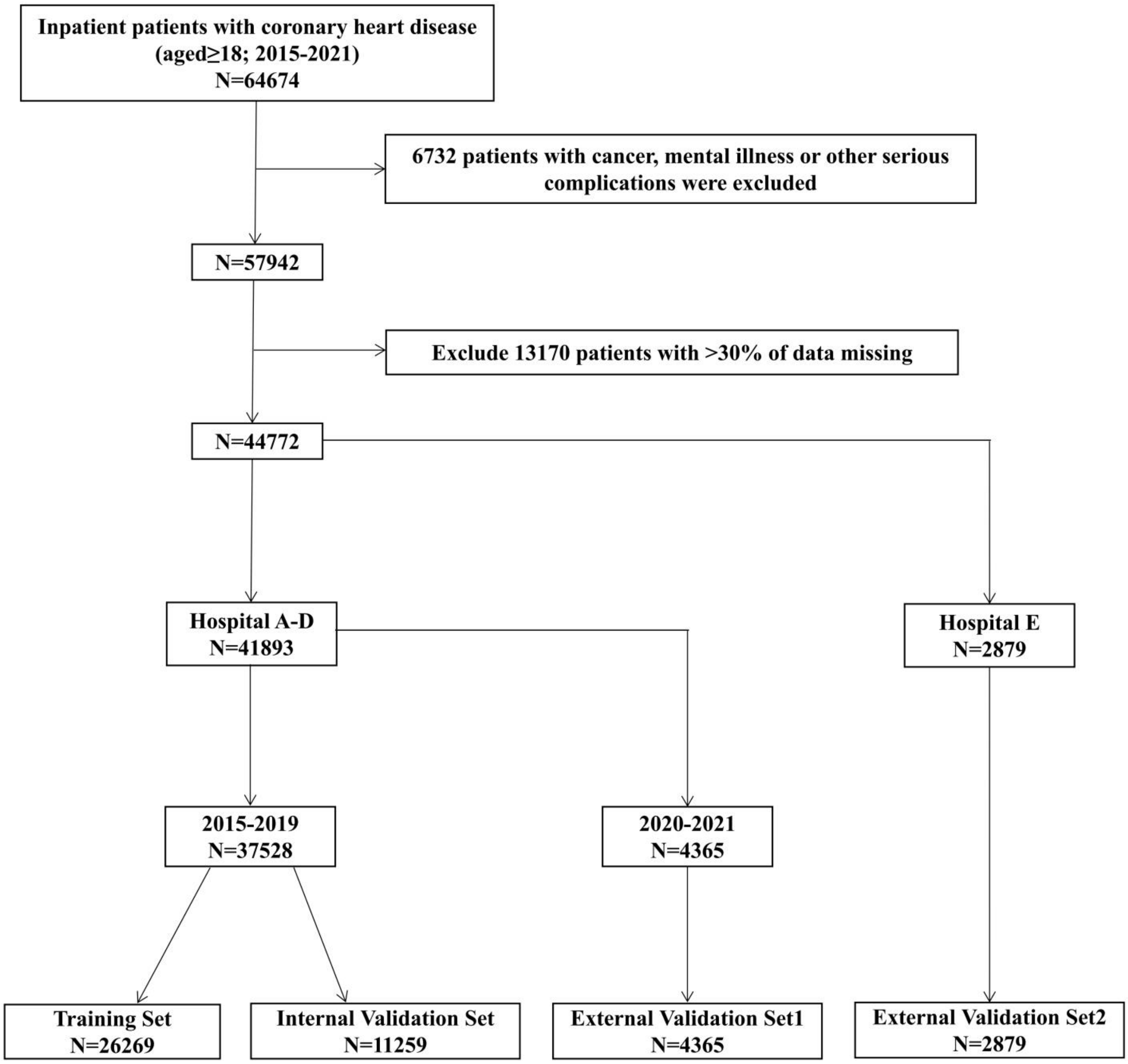

Table S1 Comparison of continuous variables in external validation set1 before and after multiple imputation

Variables	Before interpolation	After interpolation	P values
DBP (IQR, mmHg)	80.00(71.00,90.00)	80.00(71.00,90.00)	0.927
GGT (IQR, IU/L)	27.24(18.00,47.00)	27.28(18.00,48.00)	0.815
WBC (IQR, $\times 10^9$/L)	6.61(5.27,8.48)	6.61(5.26,8.55)	0.849
NLR (IQR)	3.28(2.2,5.59)	3.29(2.19,5.65)	0.913
PLR (IQR)	138.66(102.15,196.77)	138.51(102.00,197.14)	0.932
LMR (IQR)	3.13(2.05,4.44)	3.11(2.03,4.43)	0.643

SCr (IQR, umol/l)	69.90(58.00,87.10)	70.00(58.00,87.80)	0.578
TB (IQR, umol/l)	11.00(7.90,15.15)	11.02(7.90,15.18)	0.944
UA (IQR, umol/L)	340.56(274.97,418.06)	341.00(275.10,420.00)	0.719
UN (IQR, mmol/L)	5.90(4.73,7.68)	5.92(4.74,7.70)	0.851
TP (IQR, g/L)	66.60(62.00,71.20)	66.60(61.92,71.20)	0.796
ALP (IQR, IU/L)	76.80(63.00,94.00)	76.78(62.82,94.00)	0.972
LDL (IQR, mmol/L)	2.42(1.87,3.01)	2.39(1.84,2.98)	0.295
HGB (IQR, g/L)	131.00(118.00,143.00)	131.00(118.00,143.00)	0.678
RBC (IQR, $\times 10^{12}$/L)	4.30(3.90,4.71)	4.31(3.90,4.71)	0.914
BG (IQR, mmol/L)	5.92(5.13,7.89)	5.91(5.11,7.89)	0.801
AST (IQR, IU/L)	22.00(17.50,30.00)	22.00(17.53,30.00)	0.656
TGs (IQR, mmol/L)	1.32(0.94,1.94)	1.31(0.93,1.92)	0.328
TC (IQR, mmol/L)	4.42(3.65,5.27)	4.40(3.65,5.25)	0.626
HDL (IQR, mmol/L)	1.27(1.04,1.54)	1.28(1.05,1.55)	0.465

DBP: diastolic blood pressure; GGT:γ-glutamyltransferase; WBC: white blood cell; NLR: neutrophil-to-lymphocyte ratio;PLR: platelet-lymphocyte ratio ; LMR: lymphocyte-to-monocyte ratio; SCr: serum creatinine; TB: total bilirubin; UA: uric acid; UN: urea nitrogen; TP: total protein; ALP: alkaline phosphatase; LDL: low-density lipoprotein; HGB: hemoglobin; RBC: red blood cell; BG: blood glucose; AST: aspartate aminotransferase; TGs: triglycerides; TC: total cholesterol; HDL:high-density lipoprotein; IQR: interquartile range.

Table S2 Comparison of continuous variables in external validation set2 before and after multiple imputation

Variables	Before interpolation	After interpolation	P values
DBP (IQR, mmHg)	80.00(70.00,89.00)	80.00(70.00,89.00)	0.855
GGT (IQR, IU/L)	26.00(17.00,47.40)	26.00(17.00,47.40)	0.901
WBC (IQR, $\times 10^{9}$/L)	6.20(5.00,8.10)	6.20(5.00,8.10)	0.944
NLR (IQR)	3.17(2.08,5.72)	3.13(2.07,5.67)	0.727
PLR (IQR)	130.56(95.56,189.45)	130.36(95.49,189.03)	0.979
LMR (IQR)	5.30(3.27,8.18)	5.50(3.40,8.46)	0.067
SCr (IQR, umol/l)	69.90(57.10,88.10)	70.20(57.20,89.30)	0.436
TB (IQR, umol/l)	10.50(7.70,14.90)	10.50(7.70,14.90)	0.933
UA (IQR, umol/L)	330.30(265.83,412.50)	330.60(265.75,413.10)	0.923
UN (IQR, mmol/L)	6.07(4.74,8.09)	6.09(4.76,8.14)	0.688
TP (IQR, g/L)	67.60(63.10,72.30)	67.70(63.10,72.30)	0.785
ALP (IQR, IU/L)	73.15(60.60,89.20)	73.30(60.60,89.20)	0.941
LDL (IQR, mmol/L)	2.26(1.73,2.87)	2.24(1.72,2.86)	0.566
HGB (IQR, g/L)	125.00(113.00,137.00)	125.00(113.00,137.00)	0.651
RBC (IQR, $\times 10^{12}$/L)	4.21(3.84,4.61)	4.21(3.83,4.61)	0.807
BG (IQR, mmol/L)	5.74(4.94,7.33)	5.78(4.94,7.40)	0.657
AST (IQR, IU/L)	21.50(17.20,30.20)	21.50(17.20,30.40)	0.791
TGs (IQR, mmol/L)	1.17(0.86,1.71)	1.16(0.85,1.70)	0.657
TC (IQR, mmol/L)	4.30(3.54,5.13)	4.27(3.52,5.10)	0.394
HDL (IQR, mmol/L)	1.27(1.05,1.53)	1.27(1.05,1.53)	0.932

DBP: diastolic blood pressure; GGT:γ-glutamyltransferase; WBC: white blood cell; NLR: neutrophil-to-lymphocyte ratio;PLR: platelet-lymphocyte ratio ; LMR: lymphocyte-to-monocyte ratio; SCr: serum creatinine; TB: total bilirubin; UA: uric acid; UN: urea nitrogen; TP: total protein; ALP: alkaline phosphatase; LDL: low-density lipoprotein; HGB: hemoglobin; RBC: red blood cell; BG: blood glucose; AST: aspartate aminotransferase; TGs: triglycerides; TC: total cholesterol; HDL:high-density lipoprotein; IQR: interquartile range.

Table S3 Demographic and clinical characteristics of training set and external validation set1

Variables	Training set (N=26269)	External validation set1 (N=4365)	P values
Diabetes (n, %)	6194(23.58%)	1100(25.20%)	0.021
Hypertension (n, %)	10161(38.68%)	2071(47.45%)	<0.001
Smoking status (n, %)	9044(34.43%)	1636(37.48%)	<0.001
Drinking status (n, %)	7103(27.04%)	1242(28.45%)	0.054
Sex (male, n, %)	13031(49.61%)	2287(52.39%)	0.001
Age (IQR, years)	70.00(61.00,78.00)	69.00(58.00,77.00)	<0.001
DBP (IQR, mmHg)	80.00(71.00,89.00)	80.00(71.00,90.00)	<0.001
GGT (IQR, IU/L)	27.00(18.00,49.00)	27.28(18.00,48.00)	0.729
WBC (IQR, $\times10^9$/L)	6.60(5.30,8.50)	6.61(5.26,8.55)	0.752
NLR (IQR)	3.37(2.24,5.93)	3.29(2.19,5.65)	0.009
PLR (IQR)	138.13(100.91,200.00)	138.51(102.00,197.14)	0.851
LMR (IQR)	3.30(2.08,4.88)	3.11(2.03,4.43)	<0.001
SCr (IQR, umol/l)	70.00(56.30,89.60)	70.00(58.00,87.80)	0.191
TB (IQR, umol/l)	10.60(7.60,14.70)	11.02(7.90,15.18)	<0.001
UA (IQR, umol/L)	334.80(268.30,414.00)	341.00(275.10,420.00)	<0.001
UN (IQR, mmol/L)	6.10(4.85,7.92)	5.92(4.74,7.70)	<0.001
TP (IQR, g/L)	67.60(62.68,72.50)	66.60(61.92,71.20)	<0.001
ALP (IQR, IU/L)	75.27(62.00,93.00)	76.78(62.82,94.00)	0.017
LDL (IQR, mmol/L)	2.32(1.78,2.93)	2.39(1.84,2.98)	<0.001
HGB (IQR, g/L)	130.00(117.00,142.00)	131.00(118.00,143.00)	<0.001
RBC (IQR, $\times10^{12}$/L)	4.30(3.88,4.70)	4.31(3.90,4.71)	0.172
BG (IQR, mmol/L)	5.95(5.10,7.91)	5.91(5.11,7.89)	0.793
AST (IQR, IU/L)	22.00(17.20,30.00)	22.00(17.53,30.00)	0.464
TGs (IQR, mmol/L)	1.27(0.92,1.84)	1.31(0.93,1.92)	0.018
TC (IQR, mmol/L)	4.27(3.56,5.06)	4.40(3.65,5.25)	<0.001
HDL (IQR, mmol/L)	1.19(0.97,1.44)	1.28(1.05,1.55)	<0.001

DBP: diastolic blood pressure; GGT:γ-glutamyltransferase; WBC: white blood cell; NLR: neutrophil-to-lymphocyte ratio;PLR: platelet-lymphocyte ratio ; LMR: lymphocyte-to-monocyte ratio; SCr: serum creatinine; TB: total bilirubin; UA: uric acid; UN: urea nitrogen; TP: total protein; ALP: alkaline phosphatase; LDL: low-density lipoprotein; HGB: hemoglobin; RBC: red blood cell; BG: blood glucose; AST: aspartate aminotransferase; TGs: triglycerides; TC: total cholesterol; HDL:high-density lipoprotein; IQR: interquartile range.

Table S4 Demographic and clinical characteristics of training set and external validation set2

Variables	Training set (N=26269)	External validation set1 (N=2879)	P values
Diabetes (n, %)	6194(23.58%)	714(24.80%)	0.150
Hypertension (n, %)	10161(38.68%)	1521(52.83%)	<0.001
Smoking status (n, %)	9044(34.43%)	919(31.92%)	0.008
Drinking status (n, %)	7103(27.04%)	637(22.13%)	<0.001
Sex (male, n, %)	13031(49.61%)	1290(44.81%)	<0.001
Age (IQR, years)	70.00(61.00,78.00)	73.00(64.00,80.00)	<0.001
DBP (IQR, mmHg)	80.00(71.00,89.00)	80.00(70.00,89.00)	0.003
GGT (IQR, IU/L)	27.00(18.00,49.00)	26.00(17.00,47.40)	0.002
WBC (IQR, $\times 10^9$/L)	6.60(5.30,8.50)	6.20(5.00,8.10)	<0.001
NLR (IQR)	3.37(2.24,5.93)	3.13(2.07,5.67)	<0.001
PLR (IQR)	138.13(100.91,200.00)	130.36(95.49,189.03)	<0.001
LMR (IQR)	3.30(2.08,4.88)	5.50(3.40,8.46)	<0.001
SCr (IQR, umol/l)	70.00(56.30,89.60)	70.20(57.20,89.30)	0.227
TB (IQR, umol/l)	10.60(7.60,14.70)	10.50(7.70,14.90)	0.481
UA (IQR, umol/L)	334.80(268.30,414.00)	330.60(265.75,413.10)	0.276
UN (IQR, mmol/L)	6.10(4.85,7.92)	6.09(4.76,8.14)	0.925
TP (IQR, g/L)	67.60(62.68,72.50)	67.70(63.10,72.30)	0.264
ALP (IQR, IU/L)	75.27(62.00,93.00)	73.30(60.60,89.20)	<0.001
LDL (IQR, mmol/L)	2.32(1.78,2.93)	2.24(1.72,2.86)	<0.001
HGB (IQR, g/L)	130.00(117.00,142.00)	125.00(113.00,136.00)	<0.001
RBC (IQR, $\times 10^{12}$/L)	4.30(3.88,4.70)	4.21(3.83,4.61)	<0.001
BG (IQR, mmol/L)	5.95(5.10,7.91)	5.78(4.94,7.40)	<0.001
AST (IQR, IU/L)	22.00(17.20,30.00)	21.50(17.20,30.40)	0.135
TGs (IQR, mmol/L)	1.27(0.92,1.84)	1.16(0.85,1.70)	<0.001
TC (IQR, mmol/L)	4.27(3.56,5.06)	4.27(3.52,5.10)	0.752
HDL (IQR, mmol/L)	1.19(0.97,1.44)	1.27(1.05,1.53)	<0.001

DBP: diastolic blood pressure; GGT:γ-glutamyltransferase; WBC: white blood cell; NLR: neutrophil-to-lymphocyte ratio;PLR: platelet-lymphocyte ratio ; LMR: lymphocyte-to-monocyte ratio; SCr: serum creatinine; TB: total bilirubin; UA: uric acid; UN: urea nitrogen; TP: total protein; ALP: alkaline phosphatase; LDL: low-density lipoprotein; HGB: hemoglobin; RBC: red blood cell; BG: blood glucose; AST: aspartate aminotransferase; TGs: triglycerides; TC: total cholesterol; HDL:high-density lipoprotein; IQR: interquartile range.
